# Supplementary material for: PathoRM: Computational inference of pathogenic RNA methylation sites by incorporating multi-view features
Source: PLoS Comput Biol. 2025 Nov 10;21(11):e1013654. doi: 10.1371/journal.pcbi.1013654 (PMC12617926; doi:10.1371/journal.pcbi.1013654)
Supplement: S1 Text — (DOCX) [file pcbi.1013654.s001.docx]

# **Optimization Procedure of Naïve Multi-View Learning**

The objective function of multi-view learning can be decomposed into *v* independent sub-problems, where the *i*-th sub-problem is as follows.

Then we deployed Alternating Direction Method of Multipliers (ADMM) algorithm[1] to solve model (1). Given that variable ***C***(*^i^*) exhibits coupling under the ADMM framework, we introduced four auxiliary variables ***C***_1_(*^i^*), ***C***_2_(*^i^*), ***C***_3_(*^i^*)and ***C***_4_(*^i^*) in model (1) to decouple the original problem, resulting in the equivalent model as shown below:

Accordingly, the augmented Lagrangian function is defined as (3):

where $\Gamma(A,B)=<A,B>+\frac{\text{μ}}{2}||B||_{F}^{2}$, ***Y***_1_(*^i^*), ***Y***_2_(*^i^*), ***Y***_3_(*^i^*), ***Y***_4_(*^i^*) are Lagrange dual variables, and *μ* is the penalty parameter. Subsequently, we used the ADMM algorithm for solving **subproblem- *C*_1_(*^i^*)**, **subproblem- *C*_2_(*^i^*)**, **subproblem- *C*_3_(*^i^*)**, **subproblem- *C*_4_(*^i^*)**, and **subproblem-*Y***_1_(*^i^*), ***Y***_2_(*^i^*), ***Y***_3_(*^i^*), ***Y***_4_(*^i^*) distributedly and iteratively.

**subproblem- *C*_1_(*^i^*)**: with ***C***_2_(*^i^*),***C***_3_(*^i^*),***C***_4_(*^i^*),***Y***_1_(*^i^*),***Y***_2_(*^i^*),***Y***_3_(*^i^*),***Y***_4_(*^i^*) fixed, we optimized $\mathbf{L}$_c_(***C***_1_(*^i^*),***C***_2_(*^i^*), ***C***_3_(*^i^*), ***C***_4_(*^i^*), ***Y***_1_(*^i^*), ***Y***_2_(*^i^*), ***Y***_3_(*^i^*), ***Y***_4_(*^i^*)) with respect to ***C***_1_(*^i^*).

where is the singular value thresholding operator.

**subproblem- *C*_2_(*^i^*)**: with ***C***_1_(*^i^*),***C***_3_(*^i^*),***C***_4_(*^i^*),***Y***_1_(*^i^*),***Y***_2_(*^i^*),***Y***_3_(*^i^*),***Y***_4_(*^i^*) fixed, we optimized L_c_(***C***_1_(*^i^*),***C***_2_(*^i^*), ***C***_3_(*^i^*), ***C***_4_(*^i^*), ***Y***_1_(*^i^*), ***Y***_2_(*^i^*), ***Y***_3_(*^i^*), ***Y***_4_(*^i^*)) with respect to ***C***_2_(*^i^*).

where is the element-wise soft thresholding operator.

**subproblem- *C*_3_(*^i^*)**: with ***C***_1_(*^i^*),***C***_2_(*^i^*),***C***_4_(*^i^*),***Y***_1_(*^i^*),***Y***_2_(*^i^*),***Y***_3_(*^i^*),***Y***_4_(*^i^*) fixed, we optimized L_c_(***C***_1_(*^i^*),***C***_2_(*^i^*), ***C***_3_(*^i^*), ***C***_4_(*^i^*), ***Y***_1_(*^i^*), ***Y***_2_(*^i^*), ***Y***_3_(*^i^*), ***Y***_4_(*^i^*)) with respect to ***C***_3_(*^i^*).

Setting the derivate of (6) with respect to ***C***_3_(*^i^*) to zero, we can achieve the closed-form solution of ***C***_3_(*^i^*^)*^ as shown below:

**subproblem- *C*_4_(*^i^*)**: with ***C***_1_(*^i^*),***C***_2_(*^i^*),***C***_4_(*^i^*),***Y***_1_(*^i^*),***Y***_2_(*^i^*),***Y***_3_(*^i^*),***Y***_4_(*^i^*) fixed, we optimized L_c_(***C***_1_(*^i^*),***C***_2_(*^i^*), ***C***_3_(*^i^*), ***C***_4_(*^i^*), ***Y***_1_(*^i^*), ***Y***_2_(*^i^*), ***Y***_3_(*^i^*), ***Y***_4_(*^i^*)) with respect to ***C***_4_(*^i^*).

Setting the derivate of (8) with respect to ***C***_4_(*^i^*) to zero, we can achieve the closed-form solution of ***C***_4_(*^i^*^)*^ as shown below.

$$\mathbf{C}_{4}^{(i)^{*}}=(\text{μ}\mathbf{A}^{(i)^{\mathrm{T}}}\mathbf{A}^{(i)}+3\text{μ}I)^{-1}(\mathbf{A}^{(i)^{\mathrm{T}}}\mathbf{Y}_{1}^{(i)}+\text{μ}\mathbf{A}^{(i)^{\mathrm{T}}}\mathbf{A}^{(i)}+\text{μ}(\mathbf{C}_{1}^{(i)}+\mathbf{C}_{2}^{(i)}+\mathbf{C}_{3}^{(i)})-\mathbf{Y}_{2}^{(i)}-\mathbf{Y}_{3}^{(i)}-\mathbf{Y}_{4}^{(i)})$$

**subproblem-*Y***_1_(*^i^*), ***Y***_2_(*^i^*), ***Y***_3_(*^i^*), ***Y***_4_(*^i^*): the dual variables and penalty parameter *μ* can be updated with (10).

These update steps should be repeated until the following convergence constraint is met:

$\mathbf{C}_{1}^{(i)^{*}}-\mathbf{C}_{2}^{(i)^{*}}||_{\mathrm{F}}^{2}<\varepsilon,\text{ }||\mathbf{C}_{1}^{(i)^{*}}-\mathbf{C}_{3}^{(i)^{*}}||_{\mathrm{F}}^{2}<\varepsilon,\text{ }||\mathbf{C}_{1}^{(i)^{*}}-\mathbf{C}_{4}^{(i)^{*}}||_{\mathrm{F}}^{2}<\varepsilon$.

# **Message Passing and Updating Rules of Graph Neural Networks (GNNs)**

1. **Graph Convolutional Networks (GCN)**

GCN is a spectral-based graph neural network model, which can be seen as a generalized convolutional neural network. There are multiple variants of GCN, and the one adopted in PathoRM is the GCN model proposed by Kipf and Welling[2]. This model employs a first-order Laplacian approximation, simplifying the propagation rules of graph convolution, reducing the complexity of the model and maintaining algorithm performance. Its message passing mechanism is shown as (11).

$$H^{(l+1)}=\sigma(\tilde{D}^{-\frac{1}{2}}\tilde{X}\tilde{D}^{-\frac{1}{2}}H^{(l)}W^{(l)})$$

where
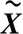
=***X*** + ***I*** represents the adjacent matrix of RM-disease heterogeneous graph with added self-loops, while
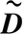
 is the diagonal degree matrix of
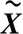
, From the node-wise view, the node embedding rule of GCN can be defined as:

where ***N*(*i*)** is the set of one-localized neighbours of node *s*_i_, $\frac{1}{\sqrt{|N(i)|}\sqrt{|N(j)|}}$ is the normalization constant.

1. **Graph Sample and Aggregation Network (GraphSAGE)**

Rather than considering all neighbours, GraphSAGE initially samples a fixed-size neighbourhood around the target node *i*[3]. Subsequently, GraphSAGE updates the node *i*’s feature embeddings by concatenating the aggregated neighbourhood embedding and its current representation by performing a direct sum operation. The updated formula for GraphSAGE can be represented as:

$$\begin{matrix} h_{N(i)}^{(l+1)}=\mathrm{MEAN}\{h_{j}^{(l+1)},\forall j\in S_{N(i)}\} \\ h_{i}^{(l+1)}=\sigma(W^{(l+1)}[h_{i}^{(l)}\oplus h_{N(i)}^{(l+1)}]) \end{matrix}$$

where MEAN denotes the mean aggregation function; ***S_N_*(*_i_*)** is the sampled immediate neighbours of node *I*; $h_{N(i)}^{(l+1)}$is node i’s aggregated neighbourhood vector; $h_{i}^{(l)}$is the node *i*‘s current representation; is the direct sum operator; and ***W***(*^l^*^+1)^ is a trainable parameter matrix.

1. **Graph Isomorphism Network (GIN)**

GIN is a GNN model designed based on the graph isomorphism theory, which is distinctly different from the GCN and GraphSAGE in that it captures the graph structure while being invariant to node feature representations[4]. Rooted in the isomorphic concept of the graph, GIN aggregates the node features by summing the aggregated neighbourhood representations, along with its own current representation, typically with a fully connected linear network and non-linear activation function, to capture complex patterns and dependencies in the graph. The feature updating formula of GIN is:

$$h_{i}^{(k+1)}=\mathrm{MLP}((1+\varepsilon^{(k+1)})h_{i}^{(k)}+\sum_{j\in N(i)} h_{j}^{(k)})$$

where $\varepsilon^{(k+1)}$ is a learnable scalar to extend the capacity of the trained model. MLP denotes a fully linear, connected neural network.

# **Metrics**

**ROC curve** uses the False Positive Rate (FPR) as the horizontal axis and the True Positive Rate (TPR) as the vertical axis, presenting the trade-off between TPR and FPR at a series of classification thresholds[5]. The formulas for calculating TPR and FPR are as follows:

$$TPR=Recall = \frac{TP}{TP+FN}$$

$$FPR=\frac{FP}{TP+FN}$$

where TPR, also known as **Recall**, represents the proportion of true positives that are correctly identified as positive by the model, while FPR refers to the proportion of true negatives that are incorrectly identified as positive by the model. Therefore, at different classification thresholds, a series of TPR and FPR values can be calculated, thus plotting the ROC curve. As a quantitative metric for evaluating the performance of the ROC curve, Area Under ROC curve (AUC) typically ranges from 0 to 1. A higher AUC value indicates better model performance, while an AUC value close to 0.5 indicates performance equivalent to random guessing.

**PR curve** regards Recall as horizontal axis, **Precision** as vertical axis, showing the trade-off between Precision and Recall for different threshold[6]. The formula for calculating Precision is as follows.

$$\text{Precision = }\frac{\text{TP}}{\text{TP+FP}}$$

Precision measures the true proportion of true positives among the predicted positives by the model. The PR curve is more informative in the imbalanced data setting. It focuses on the performance of the classifier on the positive class regardless of the number of negative samples. PR curve also reduces the performance of methods into Area under the PR curve (AUPR), where AUPR ranges from 0 to 1, where a higher value indicates better performance, and the baseline of AUPR is determined by the ratio of positive samples and negative samples.

**ACC** calculates the ratio of correctly classified samples among all samples by the model:

$$\text{ACC = }\frac{\text{TP+TN}}{\text{TP+FN+TN+FP}}$$

**Specificity** calculates the proportion of correctly predicted negative instances by the model:

$$\text{Specificity=}\frac{\text{TN}}{\text{TN+FP}}$$

**F1 score** comprehensively considers both Precision and Recall and is calculated as the harmonic mean of Precision and Recall score:

$$\text{F1 score = }\frac{2}{\frac{1}{\text{Precision}}\text{+}\frac{1}{\text{Recall}}}$$

# **PathoRM Outperforms the Traditional Machine Learning Models**

Here, we compared PathoRM with various traditional machine learning models for further demonstrating its effectiveness in RM-disease association prediction. Specifically, utilizing the RM site features extracted by iDNA-ABF and the disease semantic features extracted by BioBERT as inputs for downstream algorithms, various machine learning models, including Support Vector Machine (SVM), Random Forest, Decision Tree, Gradient Boosting, and MLP are used for comparative models. Taking 10-fold CV as experimental setting, **S1 Fig** illustrates the comparative results among PathoRM and the aforementioned models on both balanced and imbalanced m6ADA dataset.

As **S1 Fig** shows, distinct colours indicate different algorithms, whereas varying shapes represent different evaluation metrics. The closer the shapes are to the upper right corner, the higher the overall predictive performance of the algorithm on both balanced and imbalanced datasets. Conversely, algorithms perform poorer when the shapes are further from this corner. Based on this criterion, it can be observed that PathoRM outperforms the comparative classifiers across all evaluation metrics and scenarios. On the other hand, replacing the downstream components of PathoRM with various machine learning classifiers results in varying degrees of performance decline across metrics. Considering both balanced and imbalanced datasets, the ranking of overall performance among downstream algorithms is as follows: MLP, Random Forest, Decision Tree, Gradient Boosting, and SVM. These results suggest that the integration of multi-view subspace learning, graph autoencoder, adversarial training strategy and robust negative sampling technique constitutes a logical and effective system, contributing significantly to improving model performance.

**[S1 Fig]**

**S1 Fig** Comparison results of PathoRM and traditional machine learning models.

# **Investigating the Optimal Sequence Length for Motif Analysis**

To elucidate the impact of sequence length on model performance, we designed a series of truncated sequence inputs for PathoRM. Specifically, the original m^7^G-centered sequence (41 bp) was progressively shortened to 21 bp and 11 bp, while the original m^6^A-centered sequence (65 bp) was truncated to 35 bp and 15 bp, with each sequence centered on the respective modification site. These reconstructed sequences were then incorporated into PathoRM, followed by a comprehensive evaluation using 10-fold CV scheme. **S2 Fig** shows the mean AUC and mean AUPR varied with different sequence lengths of m^7^G and m^6^A.

**[S2 Fig]**

**S2 Fig** Model performance with different site-centered sequence lengths.

As **S2 Fig** shows, PathoRM demonstrated optimal performance on both the 41 bp m^7^G sequence and the 65 bp m^6^A sequence. However, as the sequence length decreased, there was a significant decline in both AUC and AUPR, with the most notable decrease observed in the shorter sequences. Specifically, the 11 bp m^7^G sequence and the 15 bp m^6^A sequence exhibited the poorest performance, with AUC values of 0.6135 and 0.6873, respectively, and AUPR values dropping to 0.6346 and 0.6645. This considerable reduction in performance may be attributed to the increased similarity between positive and negative samples as the sequence length decreases, making it more challenging to differentiate between them.

# **Parameterization Analysis of PathoRM**

The PathoRM framework is designed to identify unknown associations between RNA methylation sites and diseases. In the feature engineering stage, we manually extracted chemical and statistical features from RNA methylation sequences and fine-tuned only the final linear layer of iDNA-ABF (1,538 parameters, <0.002% of the full model) for contextual embedding extraction. For disease traits, GO features were manually derived and semantic embeddings were obtained via a frozen BioBERT model without any fine-tuning. Multi-source features were then integrated using a classical kernel-based multi-view learning module, which performs alignment and fusion without introducing trainable parameters. Finally, a graph autoencoder was employed to model associations and make predictions. As such, the graph autoencoder is the sole trainable and parameter-intensive component of PathoRM, and its parameters constitute the reported model size (see **S2 Table**).

# **Reference**

1. Boyd S, Parikh N, Chu E, Peleato B, Eckstein J. Distributed Optimization and Statistical Learning via the Alternating Direction Method of Multipliers. Found Trends Mach Learn. 2011;3(1):1–122. doi: 10.1561/2200000016.

2. Kipf TN, Welling M. Semi-supervised classification with graph convolutional networks. arXiv:1609.02907.[Submitted on 9 Sep 2016, last revised 22 Feb 2017] doi:10.48550/arXiv.1609.02907.

3. William L. Hamilton, RY., Jure Leskovec. Inductive Representation Learning on Large Graphs. arXiv:1706.02216.[ Submitted on 7 Jun 2017, revised 10 Sep 2018] doi:10.48550/arXiv.1706.02216.

4. Keyulu Xu WH, Jure L, Stefanie J. How powerful are graph neural networks? 2019. arXiv 1810.00826. [Submitted on 1 Oct 2018, revised 22 Feb 2019] doi:10.48550/arXiv.1810.00826.

5. Fawcett T. An introduction to ROC analysis. Pattern Recognition Letters. 2006;27(8):861-74. doi: 10.1016/j.patrec.2005.10.010.

6. Saito T, Rehmsmeier M. The Precision-Recall Plot Is More Informative than the ROC Plot When Evaluating Binary Classifiers on Imbalanced Datasets. PLoS One. 2015;10(3):e0118432. doi:10.1371/journal.pone.0118432. PMID: 25738806.
