# Supplementary figures and images for: PathoRM: Computational inference of pathogenic RNA methylation sites by incorporating multi-view features
Source: PLoS Comput Biol. 2025 Nov 10;21(11):e1013654. doi: 10.1371/journal.pcbi.1013654 (PMC12617926; doi:10.1371/journal.pcbi.1013654)

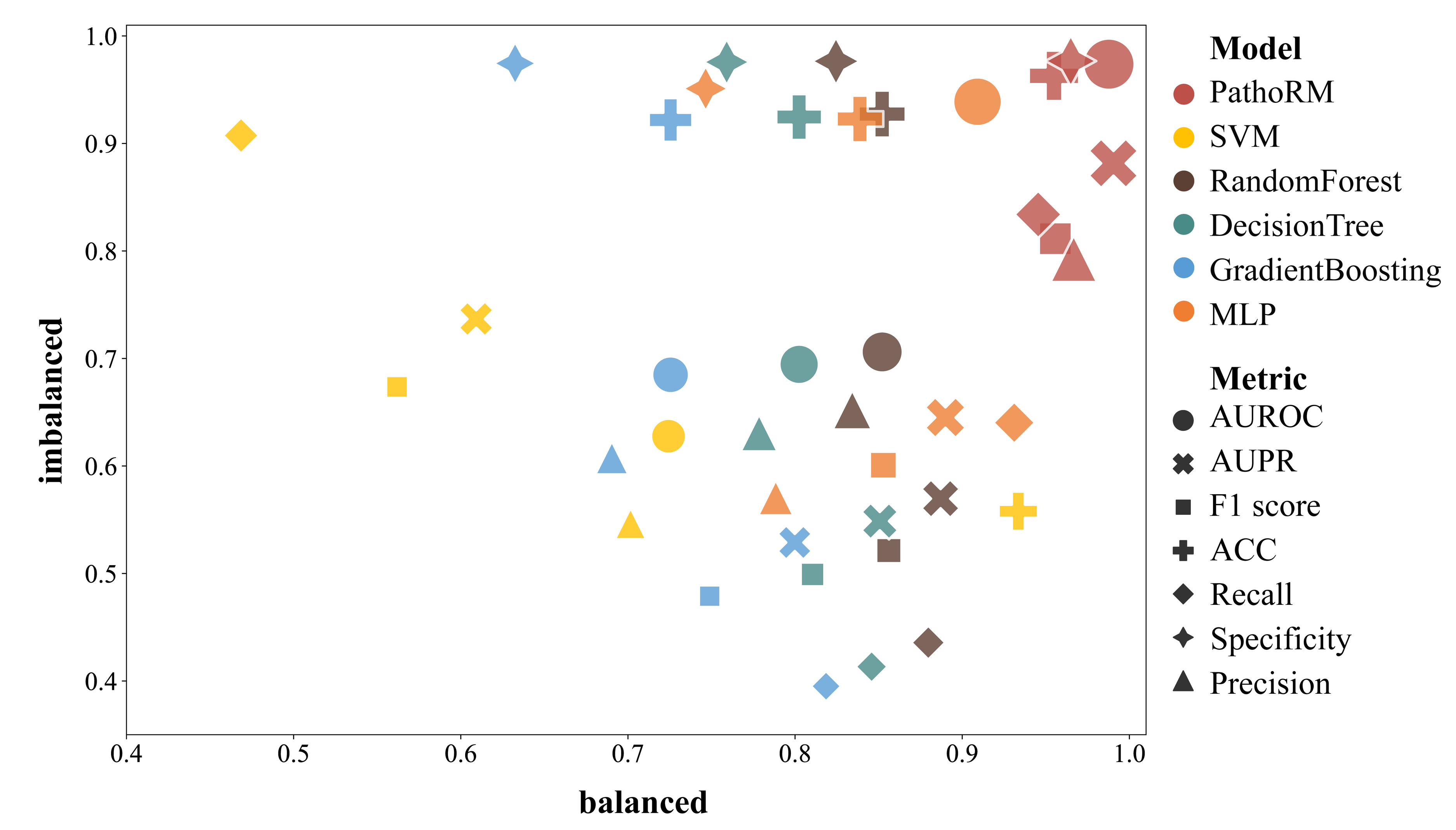

Supplement: S1 Fig — (TIF) [file pcbi.1013654.s002.tif]

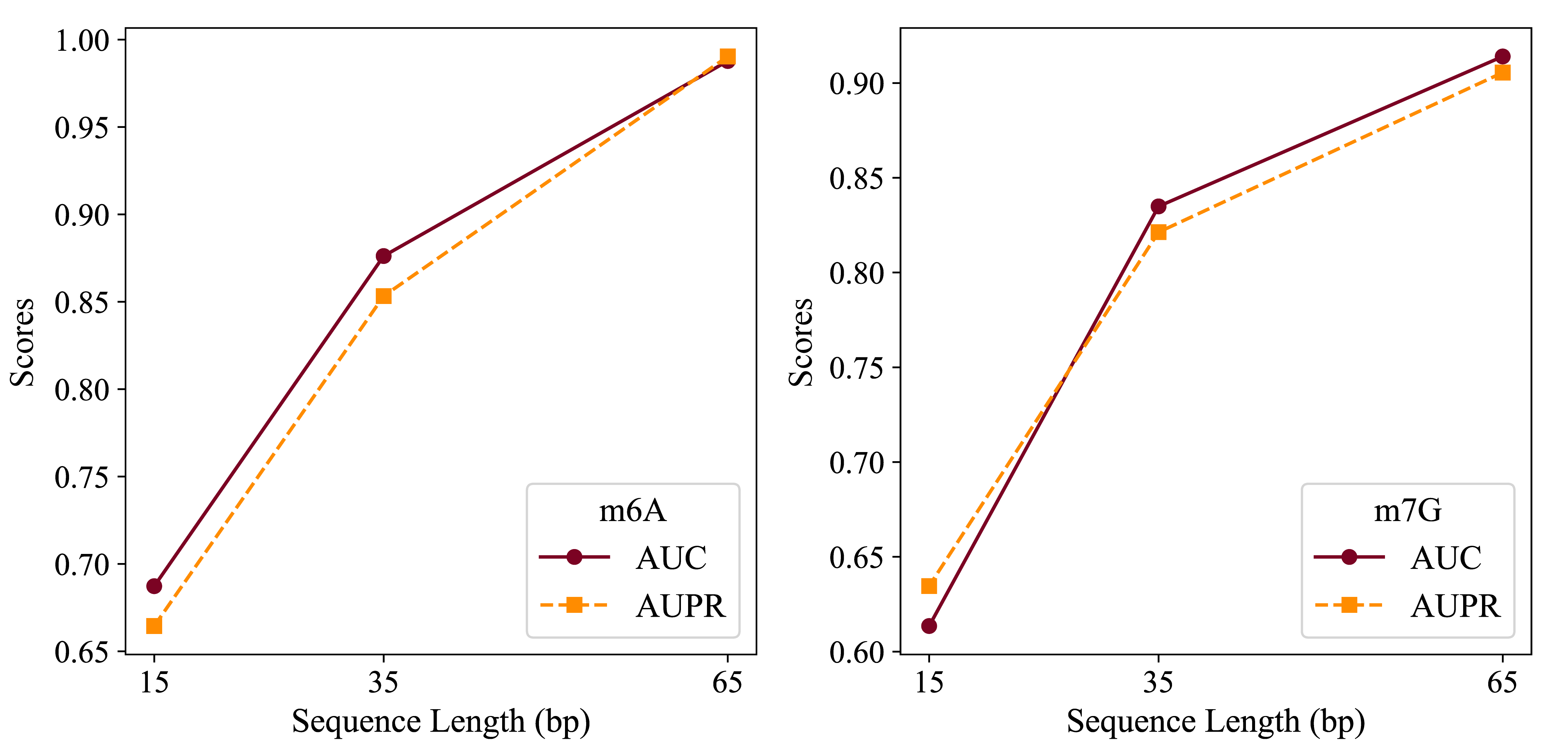

Supplement: S2 Fig — (TIF) [file pcbi.1013654.s003.tif]
