# Supplementary material for: PathoRM: Computational inference of pathogenic RNA methylation sites by incorporating multi-view features
Source: PLoS Comput Biol. 2025 Nov 10;21(11):e1013654. doi: 10.1371/journal.pcbi.1013654 (PMC12617926; doi:10.1371/journal.pcbi.1013654)
Supplement: Attachment — Submitted filename: Rebutal-Letter-PLosCB.docx [file pcbi.1013654.s007.docx]

**PathoRM: Computational Inference of Pathogenic RNA Methylation Sites by Incorporating Multi-view Features**

Hui Liu, Jiani Ma, Xianjun Ma and Lin Zhang

We are grateful to the associate editor and the reviewers for putting in efforts to review the paper to improve the quality of our paper. We have tried to address the concerns of the editor and reviewers in the revised manuscript. In particular, the following revisions have been made.

**Point-to-Point-Response**

**Reviewer 1 comments:**

1. The methods presented in this paper are highly complex, and the workflow is quite complicated. Therefore, the data calculations and variable symbols used in the algorithm are recommended to be consistently applied throughout the paper. Additionally, some symbols were not explained by the authors, which caused significant confusion for the readers. For example, $\hat{\boldsymbol{X}_{\boldsymbol{SD}}}$**,** ***H*(*l*)** and **L_WCE_**, require further clarification.

**Response:** Thank you for your valuable feedback. In response to your comments, we have carefully reviewed the entire manuscript and ensured that all data calculations and variable symbols are consistently applied throughout the paper. Additionally, we have provided explicit explanations for symbols such as$\hat{\boldsymbol{X}_{\boldsymbol{SD}}}$, ***H*(*l*)** and **L_WCE_** to enhance clarity and readability. Specifically, $\hat{\boldsymbol{X}_{\boldsymbol{SD}}}$is the predicted RM-disease association matrix, ***H*(*l*)** denotes the feature embedding of GNN encoder at the *l*-th layer. L_WCE_ refers to "Weighted Cross Entropy Loss". The corresponding descriptions have been supplemented in the manuscript. Here are the details:

**Line253:** “The predicted RM-disease association matrix $\hat{\boldsymbol{X}_{\boldsymbol{SD}}}$ is obtained by the decoder”

**Line265:** “…. parameter, and ***H***^(^*^l^*^)^ denotes the feature embedding of GNN encoder at the *l*-th layer.”

**Line356:** “where weighted cross entropy loss **L_WCE_** was applied to quantify the difference between…”

2. I recommend that the authors provide additional clarification regarding the data used in this study. Exploring and experimentally validating the effects of varying data lengths would be beneficial. Specifically, for the RNA methylation data employed in the manuscript, further details on the length of the RNA sequences and the precise location of the RNA methylation sites are necessary. Moreover, the authors should investigate whether the length of the RM sequences impacts the predictive performance.

**Response:** Thank you for your valuable and insightful comment. Based on your suggestion, we have made the necessary adjustment to the data collection and processing section, providing a more detailed explanation of the data processing procedure. The sequences used in our study are not raw sequencing data but are extracted from m7GHub and RMVar, where m7GHub provides standardized 41-bp m7G-centered reference and alternative sequences, and RMVar provides 65-bp m6A-centered reference and alternative sequences. We have carefully considered performing the length impact experiment by truncating sequences to shorter lengths, as you suggested.

To investigate the impact of sequence lengths, we systematically truncated the original m^7^G-centered (41 bp) to 21bp and 11bp, the original m^6^A-centered (65bp) to 35bp and 15bp, while preserving site-centric alignment. These variants were fed into PathoRM and assessed via 10-fold cross-validation. For your convenience, the results have been listed below.

**Fig.S2** Model performance with different site-centered sequence lengths.

PathoRM demonstrated optimal performance on both the 41 bp m^7^G sequence and the 65 bp m^6^A sequence. However, as the sequence length decreased, there was a significant decline in both AUC and AUPR, with the most notable decrease observed in the shorter sequences. Specifically, the 11 bp m^7^G sequence and the 15 bp m^6^A sequence exhibited the poorest performance, with AUC values of 0.6135 and 0.6873, respectively, and AUPR values dropping to 0.6346 and 0.6645. This considerable reduction in performance may be attributed to the increased similarity between positive and negative samples as the sequence length decreases, making it more challenging to differentiate between them. The experiments and results have been supplemented to the subsection 3.10 *PathoRM Associates Pathology with Conserved Motif* and *Investigating the Optimal Sequence Length for Motif Analysis* section in S1 Appendix.

Additionally, the supplemented description in Data collection and processing section has been listed below.

**Line 143-149:** Taking m7GDA dataset as an example, the identification of pathogenic m^7^G sites is determined by whether disease-associated genetic variants result in the gain or loss of these sites. Leveraging 1218 variant sites as a bridge, we compiled 741 pathogenic m^7^G sites with high or medium confidence level, covering 177 diseases and 768 validated associations. Additionally, each pathogenic m^7^G site is represented by a pair of 41-bp sequences—one reference and one alternative—centered on the site, ensuring comprehensive site characterization. Similarly, m6ADA consists of 2860 associations among131 pathogenic m^6^A sites and 1338 disease. For each m^6^A site, a 65-bp reference sequence and a 65-bp alternative sequence centered on the site are provided.

3. The authors should consider rephrasing some of the text in the abstract and introduction section. For example, the sentence "Here, motivated by this profound meaning, we developed a biologically informed deep learning model, named PathoRM, to help infer the associations among RNA methylation sites and diseases, providing convincing pathogenic RNA methylation sites and unraveling the enigma of pathology in the epi-transcriptomic layer” is quite long and can be split.

**Response:** Thanks for your suggestion. We have carefully revised the sentence to improve clarity and readability. The rephrased sentence is shown below.

**Line 24-27:** Here, motivated by this profound meaning, we developed PathoRM, a biologically informed deep learning model, to infer associations between RNA methylation sites and diseases. PathoRM could provide convincing pathogenic RNA methylation sites and unravel the enigma of pathology in the epi-transcriptomic layer.

4. The authors released their code on GitHub but the repository lacks extensive documentation and instructions on how to run. I expect a repository with a well-explained readme and a sh script to install a virtual env where I can run the model.

**Response:** Thanks for your valuable comment. We have supplemented the README.md file on github (<https://github.com/jianiM/PathoRM>) with detailed description of Dataset, environment preparation and step-by-step usage of PathoRM.

5. Figure 8 needs to be reformatted. In some sub-figures, the red bounding boxes interfere with the xticks, while some are overlapped with the bars.

**Response:** Thanks for your valuable comment. We have carefully adjusted the formatting of Figure8 to ensure clear illustration and improved readability. The revised figure is shown below.

6. The authors are recommended to read through the paper carefully to better improve the presentation.

**Response:** Thanks for your suggestion. We appreciate you feedback and carefully reviewed the revised paper to ensure clarity and coherence.

**Reviewer 2 comments:**

1.Dataset: The manuscript utilizes m7G and m6A as surrogates for RNA methylation in subsequent experiments. Could the authors elaborate on the rationale for selecting these two over others such as m1A, m5C, and PSI etc.?

**Response:** Thank you for your insightful question. We selected m^7^G and m^6^A as surrogates for RNA methylation in this study due to their well-documented roles in regulating disease-related pathways and the availability of abundant publicly accessible datasets, enabling robust analysis. While other modifications, such as m^1^A, m^5^C, and Ψ, are also biologically significant, their data availability and pathogenic annotations are relatively limited, making them less suitable for our study. In the future, as more data on other pathogenic RNA methylation sites become available, they can be incorporated into our research.

2. 10-fold CV: Two scenarios, balanced setting and imbalanced setting, were applied to validate the prediction performance of PathoRM. How do authors generate balanced dataset in the experiment?

**Response:** Thanks for your comment. We generate the balanced dataset in the following manner. Given on existing known positive association sample, we used our proposed “guilty-by-association”-derived negative sampling scheme, to generate one corresponding negative sample from candidate ones, thus, generating the balanced dataset where the size of negative samples is equal with the number of positive samples.

3. Setpup: In Experiments, what kind of hardware facilities can support the operation of this article's experiment. Please explain in the experimental setup and environment preparation.

**Response:** Thanks for your comment. All models in this manuscript were trained on an NIVIDA 4090 with 24GB of memory. And here are the steps for preparing environment for PathoRM.

- conda create -n bio python=3.10
- source activate bio
- pip3 install torch torchvision torchaudio --extra-index url <https://download.pytorch.org/whl/cu116>
- pip install pyg_lib torch_scatter torch_sparse torch_cluster torch_spline_conv torch_geometric -f <https://data.pyg.org/whl/torch-1.13.0+cu116.html>
- pip install torch-geometric
- pip install transformers torch

The explanation of the hardware setup and environment preparation steps has been added to the README.md file and uploaded to GitHub.

4. Figures: Figure 8 lacks consistency and alignment in the annotations of its subplots. Some of the bounding boxes overlap with the xticks, while others are positioned directly on the bars of the bar plot. Fig. 2 describes the “guilty-by-association”-based negative sampling scheme, which is good and informative. However, the figure’s caption must be written accurately and provided with a concise and useful explanation of the procedure.

**Response:** Thanks for your comment. We have carefully adjusted the formatting of Figure8 to ensure clear illustration and improved readability. The revised figure is shown below.

Additionally, we would like to respectfully clarify that Fig. 2 does not describe the “guilty-by-association”-based negative sampling scheme; rather, it presents the results of the comparison experiments. Given this, we believe the current figure and its caption are appropriately aligned with the content. However, we sincerely appreciate your feedback and would be happy to make further refinements if needed.

5. Symbolic Notation In the “3 Implementation and Optimization“ section, what does Lwce represents? Please specify the meaning of it. In the “Adversarial Training Strategy for Model Optimization” section, the authors don’t specify the meaning of H(l)

**Response:** Thank you for your valuable feedback. In the latest version, we have provided explicit explanations for symbols **L_WCE_**  and ***H*(*l*)** to enhance clarity and readability. Specifically, **L_WCE_** refers to weighted cross entropy loss. ***H*(*l*)** denotes the feature embedding of GNN encoder at the *l*-th layer. The corresponding descriptions have been supplemented in the manuscript. Here are the details:

**Line265:**“…. parameter, and ***H***^(^*^l^*^)^ denotes the feature embedding of GNN encoder at the *l*-th layer.”

**Line356:** “where weighted cross entropy loss **L_WCE_** was applied to quantify the difference between…”

6. Writing: The manuscript is generally well-structured, but there are a few long sentences that could be broken down for improved clarity and readability. For example, the sentence “Given the limitations in identifying pathogenic RNA methylation sites with in vitro experiments, there has been a need to develop computational workflow for the accurate inference of pathogenic RNA methylation to underpin biological investigations.” is lengthy. Splitting it into multiple sentences may be useful and easy to read.

**Response:** Thanks for your valuable comment. We appreciate your suggestion regarding sentence clarity and have carefully reviewed the manuscript, especially the abstract section, to refine lengthy sentences for improved readability. For example, we replaced the original sentence “Given the limitations in…” into a simpler and readable one “Due to the limitations of in vitro experiments in identifying pathogenic RNA methylation sites, there is a growing need for computational workflows to enable accurate inference.” Additionally, we splitted the long, length sentence “Here, motivated by this profound meaning, we developed a biologically informed deep learning model, named PathoRM, to help infer the associations among RNA methylation sites and diseases, providing convincing pathogenic RNA methylation sites and unraveling the enigma of pathology in the epi-transcriptomic layer” into a readable one “Here, motivated by this profound meaning, we developed PathoRM, a biologically informed deep learning model, to infer associations between RNA methylation sites and diseases. PathoRM could provide convincing pathogenic RNA methylation sites and unravel the enigma of pathology in the epi-transcriptomic layer.”

7. Equations: Some of the inline equations added to the manuscript need to be re-formatted.

**Response:** Thank you for your feedback. We have carefully reviewed the inline equations in the manuscript and have reformatted them as necessary to ensure consistency and clarity.

**Reviewer 3 comments:**

1. Biological Relevance and Background:

Practical Applications of PathoRM: While PathoRM shows promise, it would be helpful to include real-world applications or case studies, showing its utility in disease contexts. This would make the work more relevant to readers outside the computational field.

**Response:** Thank you for your insightful suggestion. We appreciate the importance of demonstrating real-world applications to broaden the impact of PathoRM. However, our study is specifically designed to investigate pathogenic RNA methylation sites, with a primary focus on identifying RNA methylation site-centered sequence motifs using an attention mechanism. This methodological approach aims to uncover sequence characteristics and potential regulatory patterns, rather than directly exploring disease context applications. While real-world case studies are beyond the scope of the current work, we recognize their value and plan to explore this direction in future research. We sincerely appreciate your thoughtful feedback and will take it into consideration as we further develop our work.

2. RNA Methylation Background: A more thorough introduction to RNA methylation and its biological implications is recommended before discussing the computational methods. This would make the study accessible to a broader audience, including those unfamiliar with deep learning.

**Response:** Thanks for your kind reminder. We appreciate the importance of providing a thorough introduction to RNA methylation. However, as the primary focus of our manuscript is to develop a computational pipeline for identifying pathogenic RM sites, we aim to keep the discussion centered on the computational aspects. In the first paragraph, we have already introduced the biological roles, functions, and implications of RNA methylation to provide essential context. Given this, we believe the current level of background information is appropriate for the scope of our study.

3. Complexity of Model Architecture: The manuscript lacks clear justifications for the many model components, such as the adaptation of iDNA-ABF (originally for DNA methylation) and the use of heterogeneous GNN structures with adversarial training. Since DNA and RNA methylation are distinct processes, further explanation is needed on why the iDNA-ABF module applies to RNA methylation.

**Response:** Thank you for your thoughtful suggestion. In my view, the application of iDNA-ABF to RNA methylation site-level feature extraction is justified for the following reasons:

**(1) Proven Effectiveness:** iDNA-ABF has demonstrated exceptional accuracy across 17 datasets, covering three methylation types, 13 species, and three human cell line datasets, highlighting its robustness and ability to extract precise methylation site features.

**(2) Model Architecture and Task Alignment:** iDNA-ABF is built upon the BERT architecture and is driven by a masked site prediction task. This design enables the model to effectively capture contextual dependencies in nucleotide sequences, a capability that is transferable to RNA methylation site prediction. Both DNA and RNA methylation processes are influenced by specific sequence motifs, recognized by methyltransferases or other regulatory proteins. While DNA methylation typically occurs at CpG sites, RNA methylation involves modifications at specific RNA sequence contexts, such as N6-methyladenosine. The language model, having been trained on DNA methylation, can learn to identify these critical sequence motifs, and with fine-tuning, the same approach can be adapted to RNA sequences and their specific methylation motifs.

Building on these points, we have incorporated and expanded the explanations within the manuscript in the subsection “2.3 LLMs for RM Sites and Disease Features Extraction”. For your convenience, the supplemented description has been listed below.

**Line 175-177:** While DNA methylation and RM are different procedures, both rely on the recognition of specific sequence motifs by regulatory proteins or enzymes. This commonality allows the model to transfer its feature extraction capabilities. Thus, driven by the task of predicting RM sites, as depicted in Fig. 1A, we retrained and fine-tuned iDNA-ABF on m6ADA and m7GDA dataset to derive contextual information of RM sites.

Additionally, adversarial training with heterogeneous GNN structures has been proved to enhance the robustness of our proposed model. The hints can be found in **Fig.6E**.

4. Adversarial Training and Negative Sampling: Both the adversarial training approach and the attention mechanism for normalization need more detailed explanations or illustrations to clarify their role in the model. This would improve the clarity of the computational approach.

**Response:** Thank you for your insightful comments. Here, we provide a more detailed explanation of the roles of adversarial training and the attention mechanism in our model. Adversarial training enhances the robustness of deep learning models by incorporating adversarial examples, which are deliberately perturbed inputs intended to induce misclassification. By learning to correctly classify such inputs, the model develops stronger defense mechanisms and improves its resistance to adversarial attacks.

In this manuscript, adversarial training is employed in PathoRM to enhance model robustness and improve generalization performance. By introducing adversarial perturbations at the GNN layer, the model learns to resist minor variations in input data, thereby mitigating overfitting. Fundamentally, adversarial training enhances the model’s ability to distinguish between genuine patterns and noise, ensuring stable feature extraction in the presence of slight perturbations. To provide readers with a clearer understanding of adversarial training, we have included a more detailed explanation in the revised edition. For your convenience, the revised part is shown below.

**Line 258-259:** Adversarial learning is a neural network regularization algorithm aimed at improving robustness against small perturbations in inputs. It achieves this by generating adversarial examples—inputs intentionally modified to maximize the model’s prediction error—and incorporating them into the training process.

Additionally, the effectiveness of the combination of adversarial training and GNN can be found in **Fig. 6E**.

**Fig. 6E** Loss curves generated by PathoRM with and without adversarial training

PathoRM incorporating adversarial training exhibits lower and more stable losses, whereas the version without it suffers from higher fluctuations, indicating instability. These results demonstrate that adversarial learning contributes significantly to improving the model’s robustness and generalization.

Additionally, the Python implementation of adversarial training in combination with GNNs can be found in our GitHub repository <https://github.com/jianiM/PathoRM>.
The attention mechanism in PathoRM is primarily derived from large-scale protein language models, specifically iDNA-ABF and BioBERT. In general, attention mechanisms dynamically assign weights to different sequence elements, allowing the model to focus on the most informative regions. This mechanism enhances feature extraction by emphasizing crucial patterns in sequence data. In PathoRM, we leverage the extracted attention weights to identify and characterize pathogenic conserved motifs, rather than merely using attention for feature transformation. This enables the model to highlight biologically significant regions that contribute to pathogenicity, providing insights into conserved sequence patterns. We hope this response clarifies the role of adversarial training and the attention mechanism in PathoRM.

5 Data Selection and Imbalance: Focus on Specific RNA Modifications (m6A and m7G): It would be helpful to explain the decision to focus on m6A and m7G modifications, given the data imbalance between the two. Disparities in sequence length (m7G: ~40 bp; m6A: ~64 bp) may affect feature quality and could explain why m6A outperforms m7G in cross-validation experiments. A more detailed justification here would strengthen the manuscript’s data approach.

**Response:** Thank you for your insightful comments. We appreciate the reviewer’s insightful comment. The superior performance observed on the m6ADA dataset compared to the m7GDA dataset is likely influenced more by differences in data density rather than sequence length alone. Specifically, the positive sample density in the m6ADA dataset is 1.632%, whereas in the m7GDA dataset, it is only 0.586%. This imbalance may lead to differences in model learning effectiveness and classification performance.

To further investigate the impact of sequence length on model performance, we have conducted additional experiments, the details of which have been incorporated into the revised manuscript. Specifically, we systematically truncated the original m^7^G-centered (41 bp) to 21bp and 11bp, the original m^6^A-centered (65bp) to 35bp and 15bp, while preserving site-centric alignment. These variants were fed into PathoRM and assessed via 10-fold cross-validation. For your convenience, the results have been listed below.

PathoRM demonstrated optimal performance on both the 41 bp m^7^G sequence and the 65 bp m^6^A sequence. However, as the sequence length decreased, there was a significant decline in both AUC and AUPR, with the most notable decrease observed in the shorter sequences. Specifically, the 11 bp m^7^G sequence and the 15 bp m^6^A sequence exhibited the poorest performance, with AUC values of 0.6135 and 0.6873, respectively, and AUPR values dropping to 0.6346 and 0.6645. This considerable reduction in performance may be attributed to the increased similarity between positive and negative samples as the sequence length decreases, making it more challenging to differentiate between them. The experiments and results have been supplemented to the subsection 3.10 *PathoRM Associates Pathology with Conserved Motif* and *Investigating the Optimal Sequence Length for Motif Analysis* section in S1 Appendix.

6.Interpretability and Scope:

Biological Interpretability: The manuscript mentions attention-based interpretability, but further detail on how attention scores correlate with known biological motifs or functions would make this clearer. For instance, validation of enriched motifs associated with diseases like breast cancer or Alzheimer’s against literature or known pathways would enhance interpretability.

**Response:** We sincerely appreciate the reviewer’s insightful suggestion. In our study, we employed pathoRM for conserved motif identification, utilizing its attention mechanism to compute attention weights for each site. This enabled us to identify high-probability distribution regions that overlap with conserved motifs detected by STREME from known disease-associated m^7^G sequences. Our aim was to illustrate this alignment as an indicator of biological relevance. However, we have not yet found literature explicitly linking the enriched motifs to Alzheimer’s or breast cancer. We recognize this as an important avenue for further investigation and appreciate the reviewer’s suggestion for enhancing interpretability.

7.Prediction Scope: The model's predictions are limited to known RM sites and diseases, preventing novel discoveries. The term “de novo prediction” might mislead readers, as it suggests new site or disease identification.

**Response:** Thanks for your kind suggestion. Based on your suggestion, we have revised the term “de novo prediction” to more accurately reflect the LODOCV experimental framework and have made the corresponding modifications in the manuscript. For your convenience, we have listed the revised part below.

**Line 299:** (iii) performance in Leave-One-Disease-Out-Cross-Validation (LODOCV) setting.

8.Presentation and Structure: Clarity of Presentation: Abbreviations, formula components, input/output structure, and certain translation issues detract from clarity. We recommend careful proofreading to improve readability.

**Response:** Thanks for your suggestion. We have carefully reviewed the manuscript to address the issues you mentioned. Additionally, we have adjusted the overall structure of this manuscript, particularly in the Results section, to improve readability. Further details of the adjustments of structure are outlined in the following comment.

9.Results Section Organization: A more hierarchical and optimized structure in the Results section would improve flow, making findings easier to follow.

**Response:** Thank you for this suggestion. We have reorganized and streamlined the Results section accordingly. Specifically, we merged the “Pattern Recognition Ability” section with the “Comparison with Traditional Machine Learning” section under the “10-fold CV experiments” section. The detailed analysis of the comparison with traditional machine learning models has been moved to the S1 Appendix. This restructuring was made because all these experiments were conducted within the 10-fold CV setting, and the primary goal was to demonstrate PathoRM’s predictive superiority and the underlying reasons for its performance advantages.

10.Comparative Analysis: Comparisons with unrelated models (e.g., drug-target and miRNA-disease models) are less relevant, while Figure 6 could be enhanced to better illustrate performance differences.

**Response:** Thanks for your comment. PathoRM is fundamentally designed to address association prediction tasks. While methods such as DeepConv-DTI, DeepDCA, and DeepDTA were originally developed for drug-target association prediction, they are well-established models in the bioinformatics field for solving association prediction problems and are therefore highly relevant to our study. Additionally, PathoRM is a deep learning framework, whereas most existing RNA methylation-disease association prediction algorithms are based on machine learning and convex optimization models. As a result, we selected DeepConv-DTI, DeepDCA, and DeepDTA as comparison methods. Furthermore, we have also included comparisons with various traditional machine learning algorithms to provide a more comprehensive evaluation.

11.Expanded Validation of Motifs:
We recommend extending motif validation to more diseases and considering recent literature on RNA methylation patterns. Additionally, highlighting novel, high-confidence predictions for validation by other researchers would further emphasize the model’s utility.

**Response:** Thank you for your valuable suggestion. We would like to clarify that the objective of the case study of conserved motif is not to identify motifs directly using PathoRM. Rather, we aim to illustrate that the high-probability regions identified by the attention mechanism in PathoRM show a significant overlap with conserved motifs identified by the STREME enrichment results. As for the potential association of these motifs with diseases such as breast cancer or Alzheimer's disease, we acknowledge that there is currently no published literature directly linking these motifs to these conditions. However, this case study is intended to highlight the potential of PathoRM in identifying biologically meaningful patterns, and we hope that future studies may explore and further validate these observations.

**Reviewer 4 comments**:

1.Most sections are subsections of 3. Please structure the paper better, since method description, benchmarking and discussion are recommended to be separated.

**Response:** Thank you for this suggestion. we have reorganized and streamlined the Results section for improved clarity. Specifically, we merged the “Pattern Recognition Ability” section with the “Comparison with Traditional Machine Learning” section under the “10-fold CV experiments” section. Then the detailed analysis of the comparison with traditional machine learning models has been moved to S1 Appendix. This restructuring was made because all these experiments were conducted within the 10-fold CV setting, and the primary goal was to demonstrate PathoRM’ s predictive superiority and the underlying reasons for its performance advantages. Overall, the Results section now includes comparison experiments (10-fold CV and LODOCV), ablation studies to validate the effectiveness of each component of PathoRM, and a case study that provides biological context to PathoRM’ s findings.

2.Regarding section 3.4: Are compared methods take all the same inputs? It should be emphasised.

**Response:** Thanks for your comment. The compared methods all take the same inputs. However, the data structure of disease traits is fundamentally different from that of drug or protein sequences, which can be directly encoded by sequence elements. To align with PathoRM, the three comparative models use a combination of GO features and embeddings generated by BioBERT as input embeddings for diseases. Then they concatenate RM chemical features, statistical features, and embeddings generated by iDNA-ABF as input embeddings for RM sites.

3.Figure 1: Diagram for the methods compared and enumerated in section 2.2 is insufficient. One should also add a dataflow diagram with blocks for the methods described in the diagram. Such dataflow diagrams for other methods in comparison should be also added.

**Response:** Thank you for your thoughtful suggestion. We would like to clarify that Figure 1 illustrates the overall workflow of PathoRM, highlighting the key modules involved in the process. The methods mentioned in section 2.2 are not comparative models, but rather the components of PathoRM itself. For example, iDNA-ABF and BioBERT serve as the semantic feature extraction modules for RM sites and disease traits, respectively. Additionally, the Native Multi-view Learning approach integrates three feature representations for RM sites and two for diseases to create an unbiased affinity matrix. This, along with the known RM-disease association matrix, is then used as input for the downstream graph autoencoder to help initialize the graph edges. As Figure 1 focuses on illustrating the PathoRM workflow, we believe it serves its purpose well.

4.Figure 2: It would be nicer to see bar-chart comparisons of each category.

**Response:** Thank you for your valuable suggestion. We understand your point regarding bar-chart comparisons. However, we believe that the spider plot offers a more comprehensive evaluation of the algorithms' performance across multiple metrics. Unlike bar charts, which present individual comparisons for each category, the spider plot enables a holistic view by displaying all metrics simultaneously. This allows for a clearer and more intuitive understanding of each algorithm's overall performance, highlighting both strengths and weaknesses in a single visualization. Additionally, it effectively showcases any trade-offs, offering a more balanced representation of the algorithms' capabilities. We hope this approach provides greater clarity and insight into the comparisons.

5.It is not clear which statistic is depicted in Figure 3 (P-value? E-value? Z-score?)

**Response:** Thank you for your valuable question. We would like to clarify that Figure 3 depicts the p-value. In the latest version of the manuscript, we have updated the figure legend to explicitly mention this detail for clarity. For your convenience, the supplemented part in the latest version is listed below.

**Line 405-406:** The statistical significance of the performance differences between PathoRM and its competing methods in the 10-fold CV scheme was evaluated using the Wilcoxon test, with the *p* value indicating the level of significance.

6.Since adversarial training scheme is attributed most of the performance, it would be nice to expand discussion and provide a simple pseudocode description.

**Response:** Thanks for your valuable comment. To provide a clearer explanation of the adversarial training approach and its underlying principles, we have expanded the discussion and added further details in the latest version:

**Lines 257-259:** Adversarial learning is a neural network regularization algorithm aimed at improving robustness against small perturbations in inputs. It achieves this by generating adversarial examples—inputs intentionally modified to maximize the model’s prediction error—and incorporating them into the training process.

Additionally, we have made the implementation of adversarial training with GNNs (including GCN, GIN, and GraphSAGE) available on https://github.com/jianiM/PathoRM, where detailed implementation instructions are provided.

7.Paper is light on possible generalizations. Is it because the discussed tool is not generalizing anything?

**Response:** Thanks for your comment. In addition to specific RNA modification, such as m^6^A and m^7^G, PathoRM has the potential to be applied to infer a broad range of pathogenic RNA modifications, including m^1^A, m^5^C, ψ, and others. Additionally, we envision that PathoRM could serve as a versatile tool for predicting various types of genetic modifications and interactions, such as pathogenic DNA methylation sites, miRNA-disease associations, and pathogenic non-coding RNAs. The flexibility of the PathoRM framework allows it to be adapted to different types of biological data and research areas, offering a promising approach for studying diverse epigenetic and transcriptomic modifications. The further discussion and generalization above has been supplemented to the Discussion section. For your convenience, we showed it below.

**Line 586-Line 591:** Finally, regardless of m^6^A and m^7^G modifications, PathoRM has the potential to be applied to a wide range of pathogenic RNA modifications, including m1A, m5C, ψ, and others. Furthermore, we envision PathoRM as a versatile tool for predicting various genetic modifications and interactions, such as pathogenic DNA methylation sites, miRNA-disease associations, and pathogenic non-coding RNAs. The flexibility of the PathoRM framework enables its adaptation to different types of biological data and research domains, making it a promising approach for studying diverse epigenetic and transcriptomic modifications.

8.I recommend adding a section 3.13 on new possible pathological motifs, if found, or discussion why no new motifs were found.

**Response:** We appreciate this insightful comment. Given the relatively limited sample size, defining novel conserved motifs requires careful consideration. Taking m^7^G as an example, we employed STEME to perform motif enrichment using known breast cancer-related m^7^G sequences and identified conserved motifs. Subsequently, PathoRM was used to predict potential breast cancer-associated m^7^G sites, excluding known sites and ranking the remaining unknown sites. For the top-ranked sites, which we regarded as novel breast cancer-related m^7^G sites, we further analysed high-probability regions based on attention weights. Following your suggestion, we compared these high-probability regions with the motifs enriched by STEME. A subset of regions that exhibited consistently high probabilities but did not align with known motifs identified by STEME may correspond to what you refer to as possible novel motifs. However, given the limited dataset, defining these regions as novel conserved motifs would not be rigorous. Additionally, from a completeness perspective, our findings—showing that the predicted novel m^7^G sites and their centered sequences align well with STEME-enriched motifs—provide supporting evidence for the biological relevance of PathoRM.

9.Dataset described in section 2.3 looks fragmentary. Discussion of the completeness of dataset coverage, and estimation of the gains from the expansion is required.

**Response:** We appreciate your comment. However, we would like to clarify that the dataset described in Section 2.3 is not the dataset used in our study but rather the dataset employed for pretraining iDNA-ABF. iDNA-ABF is a large language model originally trained on these datasets for site prediction tasks to capture epigenetic semantic features. In our study, we incorporated iDNA-ABF as a module within PathoRM and fine-tuned it specifically for extracting RNA methylation site semantic features. The datasets used for training iDNA-ABF can be found in its original publication (<https://doi.org/10.1186/s13059-022-02780-1>). Additionally, we have supplemented our discussion on the coverage of the dataset used in our study to provide a more comprehensive overview.

10.The Figures 5 and 6 is very interesting and well made. I recommend checking it for colour blind readers though. <https://accessibleweb.com/color-contrast-checker/>

**Response:** Thank you for your thoughtful comment and for bringing this to our attention. We conducted a contrast ratio analysis for Figures 5 and 6. For Figure 5, we found that the lowest contrast ratio was 1.1, which is not ideal for color-blind readers. To improve accessibility while maintaining visual consistency across the manuscript, we adjusted the colors in Figure 5. Specifically, we modified the performance representation of DeepDCA to #DEDBEE (light purple) to enhance readability. For Figure 6, the contrast ratios ranged from 1.3 (lowest) to 4.8 (highest). However, since Figure 6 includes graphical elements that help differentiate performance metrics, we decided to keep the colors unchanged to maintain consistency in the manuscript’s visual style. Additionally, in the latest version, Figure 6 has been moved to the supplementary materials due to the reorganization of the manuscript. For your convenience, the revised Fig5 has been listed below.

11.Bibliography should be expanded to properly reference some key terms, like:
Entire introduction lacks bibliography for several statements:
* ("...to extract...lncRNAs, and targets".
* "..., BioBERT... acquired contextual understanding."
* "naive multi-view algorithm"
* "In spite of promise of some of the approaches..." (please cite at least once per approach enumerated)
* "...adeptly handling diverse data structures like..."
* "...thereby unveiling cryptic patterns..." [不涉及参考文献]
1. Xavier initialization strategy in section 3.3.
2. "matrix-decomposition derived decoder" at the end of section 2.5. [公式2，无参考文献]
3. "PathoRM tries three GNNs, including GCN, GraphSage, and GIN."
4. Section 2.4 "CNF feature matrix" - either bibliography or expanding abbreviation on first use is required. Same for "disease GO feature matrix". (Not everybody is necessarily familiar with Gene Ontology etc.)

5. Please expand abbreviations on first use for "DOID and GWAS identifiers" in section 2.3.

**Response:** Thanks for your valuable comment. For BioBERT, naïve multi-view learning, Xavier initialization strategy, GCN, GraphSAGE, GIN and methods that are needed to be listed, we have supplemented the reference. The supplemented references have been listed below.

**Line92:** numerous models have been developed and tailored for prioritizing potential targets, drugs, pathogenic miRNAs or lncRNAs[20-22]…

**Line117:**BERT[30] trained on vast biomedical corpora have acquired contextual understanding,

**Line126:** naïve multi-view learning algorithm[32]…

**Line76:** Despite the promise of these approaches[17-19], some challenges remain for the inference of pathogenic RM sites,…

**Line353:** Xavier initialization strategy[44]…

**Line 237:** PathoRM tries three GNNs, including GCN[36], GraphSAGE[37] and GIN[38]…

Additionally, the phrases “adeptly handling diverse data structures like” and “thereby unveiling cryptic patterns” are simply descriptive expressions and do not require citations.

CNF is the abbreviation of cumulative nucleotide frequency, and only be used in the manuscript only once. Thus, in the latest version, we give its full name, such as:

**Line 199:**… represents the RM cumulative nucleotide frequency feature matrix with continuous values…

DOID and GWAS are the abbreviations of Disease Ontology Identifier and Genome-Wide Association Study. We have supplemented their full name in the latest manuscript:

**Line 185:** …based on Disease Ontology Identifier (DOID) or Genome-Wide Association Study (GWAS) identifiers.

12.Please note that missing page and line numbers make the submission somewhat inconvenient to review and correct.

**Response:** Thank you for your comment. I have added continuous line numbers in the updated version to facilitate the review process.

13.As for the code, best practices dictate adding package metadata (`requirements.txt` file with the versions of packages needed to install and tested) and CI build to the GitHub repository.

**Response:** Thanks for your valuable comment. We have supplemented the README.md file on github (<https://github.com/jianiM/PathoRM>) with detailed description of Dataset, environment preparation and step-by-step usage of PathoRM.
