# Supplementary material for: PathoRM: Computational inference of pathogenic RNA methylation sites by incorporating multi-view features
Source: PLoS Comput Biol. 2025 Nov 10;21(11):e1013654. doi: 10.1371/journal.pcbi.1013654 (PMC12617926; doi:10.1371/journal.pcbi.1013654)
Supplement: Attachment — Submitted filename: RESPONSE-TO-REVIEWERS.docx [file pcbi.1013654.s008.docx]

**PathoRM: Computational Inference of Pathogenic RNA Methylation Sites by Incorporating Multi-view Features**

Hui Liu, Jiani Ma, Xianjun Ma and Lin Zhang

We are grateful to the associate editor and the reviewers for putting in efforts to review the paper to improve the quality of our paper. We have tried to address the concerns of the editor and reviewers in the revised manuscript. In particular, the following revisions have been made.

**Point-to-Point-Response**

**Reviewer 3 comments:**

The authors have responded thoroughly to most reviewer comments, particularly around technical detail, code clarity, and model architecture. They added new analyses and reorganized the manuscript for improved readability. However, one key concern remains: the biological relevance and real-world utility of the model. As stated in the journal’s scope, PLOS Computational Biology values research that not only develops novel computational methods but also provides new biological insights or shows potential for discovery. This manuscript still lacks a concrete demonstration of how the model advances biological understanding, even in a preliminary or illustrative way. The rebuttal defers this to future work. In my view, this limits the impact of the paper within the journal’s mission. If the paper is to be accepted as a Methods or Research article, I believe a stronger emphasis on biological interpretability or a basic case study linking predicted methylation sites to a disease context would be needed. I would support publication if the editorial team feels that the methodological novelty alone meets threshold, but from my perspective, it still falls short of delivering on the biological significance that the journal prioritizes.

**Response**: Thank you very much for your thoughtful and constructive comment. We greatly appreciate your insight, and we sincerely agree that illustrating the predictions in a disease context is essential for confirming the practical biological value of our approach. However, we would like to note that a major limitation we currently face is the lack of publicly available clinical reports confirming pathogenic RM sites. In the absence of definitive clinical annotations, it is currently not feasible for us to directly validate the predicted sites against experimentally confirmed disease associations.

In light of this limitation, we have attempted to address the biological significance of our model through an alternative approach, which we present in Subsection **3.10 *PathoRM Associates Pathology with Conserved Motif****.* In this case study, we treated all validated associations (*s*, *d*)∈Ω as positive training samples while the others (*s*, *d*) ∉ Ω as candidate samples. Then we applied our trained model to score these candidates and prioritize RM sites potentially associated with specific diseases. Specifically, we focus on breast cancer and Alzheimer’s disease as representative examples, and select the top three RM sites for further biological investigation. To assess whether these predicted sites may plausibly relate to disease, we leveraged the attention mechanism within our model to compute the contribution of each nucleotide and compare the resulting high-attention regions with conserved motif derived from experimentally validated, disease-associated RM sites. As shown in **Fig.7**, the predicted high-risk regions significantly overlap with these conserved sequence patterns. These results suggest that, even in the absence of explicit clinical annotations, the proposed model exhibits a promising ability to capture intrinsic pathogenic signatures at the sequence level.

We fully acknowledge that this approach is an indirect substitute for clinical validation, and we sincerely appreciate your emphasis on the need for more biologically grounded assessments. We are committed to pursuing this direction further, and we anticipate that as more experimental or clinical data becomes available, we will incorporate such evidence to directly validate our predictions in future studies.

**Reviewer 4 comments:**

1. I really enjoyed reading this article, but I found an important omissions that prevent understanding, verification, and reproduction of the research. In particular, detailing number and width of neural network layers for each component of the PathoRM should always be reported. Standards for new AI papers require that the structure of the network is shown as a graph with each component having these numbers reported. Both dimensions, and activation functions should be reported. For a great example of such report see figures 2 and 3 of "Going deeper with convolutions" of Szegedy et al. Interesting tool for making publication quality neural network structure graphs is PlotNeuralNet of Haris Iqbal (<https://github.com/HarisIqbal88/PlotNeuralNet)>. (The only clear indication of the number of layers was found by the reviewer in line 245, and only for the GNN part. While dimensions of BioBERT may be found in another publication, leaving them out makes the PathoRM paper feel like a supplementary to BioBERT paper referenced as [31], instead of a self-contained research report.) Total size of parameters (in megabytes) should also be reported. While training procedure seems carefully references, the omission of clear and complete description of network structure would prevent reproduction of this research and falls short of PLoS requirements for the data included.

**Response:** Thank you for your valuable and insightful comment. To ensure transparency and reproducibility, we have supplemented the statistical summary of network structure (including model size and hyperparameters) in **TableS2** of **S1 Appendix**. Specifically, the proposed PathoRM framework is designed to identify unknown associations between RNA methylation sites and diseases. The overall logic of the model is as follows:

In the feature engineering process, with RNA methylation sequences as inputs, we manually extracted the chemical features and statistical features of RNA methylation sequences, and then fine-tuned iDNA-ABF for extracting the contextual embeddings for RNA methylation sites. We fine-tuned only the final linear classification layer of iDNA-ABF using the m⁶A site prediction task. This introduced only 1,538 trainable parameters (768×2 + 2 for bias), accounting for less than 0.002% of iDNA-ABF’s full parameter set. As for disease trait descriptions, we manually extracted the GO features and semantic features with BioBERT. During this procedure, BioBERT was used exclusively as a frozen feature extractor to obtain semantic embeddings for disease names. We did not fine-tune any part of the BioBERT model during training, all of its parameters were kept fixed. In the multi-view learning process, the heterogeneous features are then integrated using a multi-view learning strategy, which allows us to learn unbiased, data-driven representations. The multi-view learning module is implemented based on classical kernel-based alignment and integration methods, and does not contain trainable deep learning parameters. Its role is to align and integrate multi-source feature spaces rather than to learn high-dimensional feature transformations, hence it contributes no neural network parameters to the overall model. In the association prediction procedure, the graph autoencoder was conducted for final inference of unknown RNA methylation site-disease associations.

As such, the main parameter-intensive and trainable component of PathoRM lies in the graph autoencoder module, which is responsible for modeling associations and learning from the integrated network.

**Table S2**. Statistical summary of model hyperparameters setting and trainable parameters

|  | **Component** | **Input dimension** | **Output**  **dimension** | **Activation Function** | **Dropout rate** | **# Parameters**  **(Trainable)** |
| --- | --- | --- | --- | --- | --- | --- |
| **GCN – Graph**  **Encoder** | GCN | 918 (m7G)  1469 (m6A) | 128 | LeakyReLU | 0.3 | 0.27M (m7G)  0.41M (m6A) |
|  | GCN | 128 | 128 | LeakyReLU | 0.3 |  |
|  | GCN | 128 | 128 | LeakyReLU | 0.3 |  |
|  | Decoder | 128 | 918 (m7G)  1469 (m6A) | / | / |  |
| **GraphSAGE-GraphEncoder**  **Encoder** | GraphSAGE | 918 (m7G)  1469 (m6A) | 200 | LeakyReLU | 0.5 | 0.41M (m7G)  0.63M (m6A) |
|  | GraphSAGE | 200 | 200 | LeakyReLU | 0.5 |  |
|  | Decoder | 200 | 918 (m7G)  1469 (m6A) | / | / |  |
| **GIN-GraphEncoder** | GIN | 918 (m7G)  1469 (m6A) | 200 | LeakyReLU | 0.5 | 0.49M (m7G)  0.71M (m6A) |
|  | GIN | 200 | 200 | LeakyReLU | 0.5 |  |
|  | Decoder | 200 | 918 (m7G)  1469 (m6A) | / | / |  |

2. Comparison of PathoRM statistcs in **Fig 2A** with the sum of ideal hyperbole corrected with a regression slope to assure confirm or reject almost perfect fit of the results.

**Response:** We appreciate the reviewer’s suggestion. **Fig 2A** presents the ROC and PR curves generated by PathoRM and several baseline models. This figure aims to evaluate classification performance on the task of predicting associations between RNA methylation sites and diseases, which is inherently a binary classification task rather than a regression problem. ROC curves and PR curves are widely used to assess the effectiveness of classification models. The ROC curve illustrates the trade-off between true positive rate (sensitivity) and false positive rate, while the PR curve depicts the precision versus recall. A classifier with high performance will produce an ROC curve that approaches the top-left corner, and a PR curve that closely follows the top-right corner. These ideal lines (perfect classifier) are represented by step-wise curves: an ROC curve that reaches (0,1) immediately, and a PR curve that maintains high precision across all recall levels. In our case, the red lines in **Fig 2A** (representing PathoRM) already lie very close to these theoretical optimal lines. Adding explicit idealized step functions or regression-corrected baselines may lead to visual overlap and reduce figure clarity without providing significant additional information. Therefore, we opted not to include an additional reference curve in the plot.

3. Clearly state whether different subnetworks fine-tuned (BioBERT) or trained separately, or all together?

**Response:** Thank you for your insightful comment. We would like to clarify that PathoRM is not designed as an end-to-end model; rather, it consists of three separate stages: feature engineering, multi-view learning, and a graph auto-encoder with adversarial training. The feature engineering stage is completed independently before the latter two stages and incorporates models that are either fine-tuned or directly applied based on their original design and usage. Specifically, iDNA-ABF, which was initially developed for DNA methylation site prediction and feature extraction, was fine-tuned for RNA methylation feature extraction in the context of our task. In contrast, BioBERT, having been pre-trained on large-scale biomedical corpora and fine-tuned on downstream disease-related tasks, was directly employed to extract semantic features of diseases without further fine-tuning. To enhance clarity, we have explicitly stated the implementation details in section **2.3 LLMs for RM Sites and Disease Features Extraction** in the latest version. For your convenience, we listed the revised sections below:

2.3 LLMs for RM Sites and Disease Features Extraction

Line 177-179: Thus, as depicted in **Fig. 1A**, we fine-tuned iDNA-ABF on the m6ADA and m7GDA datasets for the task of RM site prediction, enabling the model to derive contextual sequence features associated with RNA modifications.

Line 187-188: …, and then directly applied the pre-trained BioBERT model without any further fine-tuning, keeping its parameters frozen throughout.

4. Total size of training data and validation inputs mentioned in section 2.1.

**Response:** Thank you for your valuable comment. In Section **3.1 Experimental Design**, we have explicitly described the composition of the training and test datasets. These configurations vary depending on the experimental setting, including:

- **Experimental Setting** We adopted both 10-fold CV and LODOCV. In 10-fold CV, all validated RM-disease associations were randomly partitioned into 10 subsets; each fold used 9 for training and 1 for testing, combined with sampled negative pairs. LODOCV evaluated performance under the “cold-start” setting, where one disease was held out for testing and the remaining diseases were used for training.
- **Dataset** Experiments were conducted on two datasets: m7GDA and m6ADA. m7GDA consists of 768 associations (positive samples) among 741 pathogenic m6A sites and 177 diseases, while m6ADA consists of 2860 associations (positive samples) among131 pathogenic m6A sites and 1338 diseases.
- **Training scenarios** Under the 10-fold CV scheme, we designed both balanced and imbalanced training setups. The balanced setting included an equal number of negative and positive samples, while the imbalanced setting used a 10:1 ratio of negatives to positives, reflecting real-world data imbalance.

Accordingly, we now provide a detailed summary of the training and test data sizes under each configuration. Firstly, under 10-fold CV scheme, the statistics of training set and test set across datasets and experimental settings are listed below.

|  |  | training set | | test set | |
| --- | --- | --- | --- | --- | --- |
|  |  | positive samples | negative samples | positive samples | negative samples |
| m7GDA | balanced | 691 | 691 | 77 | 77 |
|  | imbalanced | 691 | 6910 | 77 | 770 |
| m6ADA | balanced | 2574 | 2574 | 286 | 286 |
|  | imbalanced | 2574 | 25740 | 286 | 2860 |

LODOCV was designed to assess the models’ capabilities in prioritizing the pathogenic RM sites under the “cold-start” scenario. Briefly, taking disease *d_i_* as an example, the remaining diseases constitute the set D\*d_i_*. Then *d_i_* -associated validated samples (*s*, *d_i_*), *s*∈S, are regarded as test sample while validated *s*-D\ *d_i_* pairs are regarded as training set. Thus, in the LODOCV scheme, the size of training and test sets is not fixed, as it depends on the number of known disease-associated RM sites for each disease.

5. Minor issues that reviewer have also found: References for the methods used should be placed consistently after the method abbreviations instead of occasional shift to the end of the sentence. In particular, Line 98: Abbreviations DeepDTA and DeepDCA should be disambiguated with a paper reference placed immediately after each abbreviation.

**Response:** Thanks for your kind reminder. In the latest version, we have revised the manuscript to include appropriate references immediately following DeepDTA and DeepDCA at their first appearance. Similarly, references for other algorithmic models have also been placed directly after each model name to ensure clear attribution. For your convenience, the revised parts have been listed below.

Line 98: For instance, DeepDTA[23] and DeepDCA[24] respectively…

Line 101: HyperAttentionDTI[25] utilizes…

Line 104: GraphDTA[26] employs…

Line 106: DrugVQA[27] utilizes …

Line 108: KGE_NFM[28] leverages…

6. Line 139: Paragraph starts with doubly subordinate sentence using passive mode that is extremely difficult to follow.

**Response:** Thanks for your comment. We have revised the sentence to improve clarity and readability. The updated version reduces the complexity of the original structure while preserving its intended meaning.

**“** Rooted in the biological premise that genetic variation at certain loci leads to abnormal RM expression and contributes to disease development, we curated two pathogenic RM site datasets: the m7G-Disease Association (m7GDA) dataset from m7GHub [14], and the m6A-Disease Association (m6ADA) dataset from RMVar [13], for downstream model evaluation.”

7. Figure 2.1 should be attached as vector graphics.

**Response:** Thank you for your helpful comment. We fully understand the advantages of using vector graphics formats such as EPS for better scalability and clarity. However, TIFF format is generally more straightforward to work with, as EPS files often suffer from issues such as missing or corrupted fonts, oversized masks, stray points, and bounding box errors, which may cause rendering problems or poor output quality. To maintain consistency with the other figures in the manuscript and considering the aforementioned technical concerns, we have retained the figure in TIFF format. Nonetheless, to ensure that the figure content and details are presented clearly, we have increased the resolution and set a high DPI value to enhance visual quality. We hope this solution meets the production standards while maintaining clarity and consistency throughout the manuscript.

8. Minor issue:

Line 323: "AUC metric" -> "The AUC metric"
Line 324: "LOODOCV" -> "the LODOCV"
Line 435: "distribution of AUC scores" -> "distribution of AUC statistic distributions"
Line 449: Description of figure 5 should state "Distributions of AUC statistic for..." instead of generic "Comparison Performance of ..."

Line 450: The figure caption has been revised to “Distributions of AUC statistics for...” to better describe the figure.
Line 569: "Convervative motif" -> "Conserved motif"
Line 576: "positive ad negative" -> "positive and negative"

**Response**: Thank you for your valuable comments. We have carefully addressed each of the suggested issues as follows, and marked as highlight in the latest version.

- Line 322: Revised to **“the AUC metric”** for clarity.
- Line 323: Revised to **“the LODOCV scheme”** to reflect proper grammatical usage.
- Line 436: We used the expression **“distribution of AUC statistic”** to avoid redundancy, as “distribution of AUC statistic distributions” contains repeated terms. The current phrasing is widely accepted and clearer.
- Line 450 The figure caption has been revised to **“Distributions of AUC statistics for...”** to better describe the figure.
- Line 569: Corrected **“Convervative motif”** to **“Conserved motif.”**
- Line 577: Corrected **“positive ad negative”** to **“positive and negative.”**

9. Figure 5 (Lines 447–450): The Y-axis label has been updated to “distribution density” for clarity.
